# Revisiting the scope and expectations of Implementation Science and Implementation Science Communications

**DOI:** 10.1186/s13012-024-01399-z

**Published:** 2024-10-04

**Authors:** Paul Wilson, Gregory A. Aarons, Anne Sales, Dong (Roman) Xu, Michel Wensing, Alison Hutchinson, Rinad S. Beidas, Elvin Geng

**Affiliations:** 1 Centre for Primary Care and Health Services Research, University of Manchester, UK and NIHR Applied Research Collaboration Greater Manchester, Manchester, UK; 2 Department of Psychiatry, University of California San Diego, La Jolla, CA USA; 3 Sinclair School of Nursing and Department of Family and Community Medicine, University of Missouri, Columbia, MO USA; 4 Center for Clinical Management Research, VA Ann Arbor Healthcare System, Ann Arbor, MI USA; 5 Acacia Lab for Implementation Science, SMU Institute for Global Health (SIGHT), School of Health Management and Dermatology Hospital, Southern Medical University (SMU), Guangzhou, China; 6 Department for General Practice and Health Services Research, Heidelberg University Hospital, Heidelberg, Germany; 7 School of Nursing and Midwifery at Deakin University, Melbourne, Australia; 8 Department of Medical Social Sciences, Feinberg School of Medicine Northwestern University, Chicago, USA; 9 Center for Dissemination and Implementation, Division of Infectious Diseases, Washington University in St. Louis, St. Louis, USA

## Abstract

This editorial updates the scope and submission expectations of *Implementation Science* and *Implementation Science Communications*. We refine our protocol publishing policies and set out new expectations for reporting studies describing determinants and their relationship with implementation outcomes. Our central focus remains on the implementation of evidence-based interventions into healthcare practice and policy. We are most interested in rigorous empirical studies of the implementation of evidence-based healthcare interventions, practices, and policies, and the de-implementation of those that are demonstrated to be of low-value or no benefit. Alongside this, we remain interested in the systematic study of implementation mechanisms and processes and on the influences of patient, professional, and organizational behaviours. Novel theoretical and methodological developments are considered. For all submissions, we expect authors to demonstrate how their work is integrated with existing knowledge in the field and to clearly state the added value of the work to the field broadly.

## Background

In the two decades since *Implementation Science* was founded, we have witnessed a rapidly growing global interest in methods to enhance the uptake of evidence-based practices, programs, and policies that affect healthcare delivery and health outcomes in clinical, organizational, public health, or policy contexts. This interest has been fuelled by increased research funding and infrastructure and an ever-growing community of dedicated researchers and practitioners across the world. We routinely receive submissions from authors in over 100 countries and *Implementation Science* is now firmly established as one of the leading journals in Health Policy and Services (Clarivate Journal Citation Reports).

Given this growing interest in the discipline and the concomitant growth in manuscript submissions, we launched *Implementation Science Communications* in February 2020 as a companion journal to *Implementation Science*. The aim was to support the growth of the field by offering a destination for a broader variety of papers reporting on aspects of the science of implementation. Although still relatively young, *Implementation Science Communications* has grown rapidly and now receives approximately 350 submissions a year. The journal is indexed in the Emerging Sources Citation Index, PubMed, PMC and Scopus, and is due to receive its first Impact Factor from Clarivate in 2025.

Although companion journals, with collaboration across the Editors-in-Chief and Associate Editors, independent editorial decisions are made at each of the journals; transfer from one to the other does not imply that a manuscript will be accepted for publication, although the likelihood of review is higher than average for transferred manuscripts. As our editorial processes and transfer criteria have evolved, it is time to reflect on both journals’ scope so that prospective authors have a clearer understanding of which journal is best placed to consider their submission. We urge authors to consider the scope of each journal and submit manuscripts to that which is most appropriate in order to streamline the submission process and avoid delays inherent in transfer between journals.

## Scope of the journals

This editorial details some changes in scope since we last outlined our expectations in 2021 [1].

Overall, across both journals, our central focus remains on the implementation of evidence-based interventions into healthcare practice and policy. We are most interested in rigorous empirical studies (including associated process and economic evaluations) assessing the effects of deliberate and purposive actions to implement evidence-based interventions, practices and policies, and the de-implementation of those demonstrated to be of low-value or no benefit. We rarely receive, but prioritize direct comparisons of implementation strategies (e.g., comparative effectiveness implementation trials). There may be a perception that for evaluations of strategy effectiveness we are rigidly focused on randomised designs. This is not true. We consider other rigorous designs such as quasi-experimental designs, including interrupted time-series analyses, difference-in-difference, and/or natural experiments using synthetic controls. In all instances, the rationale for the design selected must be clearly articulated.

Alongside strategy effectiveness, we welcome the study of implementation contexts, mechanisms and processes and their influences on patient, professional, and organizational behaviours. Novel theoretical developments that relate to the field are also considered. In all instances, a variety of methodological approaches, including qualitative, quantitative and mixed-methods are considered.

The scope of the two journals overlap but there is some delineation in content*. Implementation Science* focuses on rigorous studies that substantially advance the field by providing innovative, analytical, and generalizable insights. *Implementation Science Communications* has a broader scope encompassing methodologically sound studies that contribute new knowledge (by extending existing concepts and methods), but may have a narrower focus in terms of clinical topic, descriptive design (e.g., identification of determinants, mechanisms, or strategies), patient population or setting.

There are now thousands of descriptive studies exploring barriers and enablers to implementation. Many submissions to the journals are single studies that lack any grounding in, or learning from this wider literature; often despite the fact that much of it has been consolidated through synthesis. There is, therefore, much duplication of effort and research waste through the presentation of isolated findings. *Implementation Science Communications* will continue to consider submissions focused on implementation determinants, but we expect these to be theory-driven, address empirical gaps, and ideally involve multiple sites or longitudinal studies, or demonstrate novelty in methods. Highly descriptive determinants papers or those with a narrower disciplinary or clinical focus will now only be considered for publication as Short Reports.

Our focus on health is broad and includes health services and systems, clinical practice, preventive care, and population health interventions delivered in traditional healthcare settings (e.g., hospitals, clinics) and other settings (e.g. schools, churches, prisons, etc.). We recognise that, as a field, implementation science benefits from, draws from, and includes other sectors and disciplines, but as an open access journal, with finite editorial and reviewer resources, we continue to focus on implementation science in healthcare.

Where our focus is evolving, or rather becoming more explicit, is in how we operationalise the concept of evidence in healthcare. We have written about this in detail elsewhere and urge authors to review our guidance [2], as we recognise that different thresholds apply to the evidence standards for population health interventions, organizational change, health reforms, health policy implementation, digital health and medical devices. Consequently, our assessment of the appropriateness of the evaluation design used to assess implementation efforts in these varied contexts will also consider these thresholds for evidence.

We are aware that many national health systems are developing more streamlined pathways to provide faster access to novel technologies and products, to improve the health care that people receive. This often means that real world implementation efforts run in tandem with regulatory approvals and technology assessments. In this context, we are interested in evaluations of systematic efforts to accelerate the diffusion and dissemination of innovations at scale in health systems. In all instances, we expect authors to provide a consolidated and synthesized summary of the relevant evidence for the object(s) of implementation.

There of course remain some boundaries. We are interested in the planned implementation of evidence-based digital health interventions and strategies to promote utilisation, but not in the technical implementation of digital infrastructure. We also consider strategies in which patients have agency and may directly influence the behaviour of healthcare professionals and or efforts to promote evidence-based practice. However, we routinely desk-reject submissions that involve only patients in their individual health behaviour change. We consider process evaluations of complex clinical or public health interventions on a case-by-case basis, and favour those conducted alongside or combined with rigorous evaluation of the effects of implementation strategies on determinants, mechanisms, and outcomes.

We remain committed to protocol publication for as this promotes information sharing and increases transparency, enabling comparison between what was initially planned and then actually done. The protocol paper also provides an early glimpse into where the field is moving in terms of empirical studies, as results are published years later. We have decided to consolidate our criteria and both journals will now only consider protocols that have been through competitive peer review to receive funding from a nationally or internationally recognised research agency and that have received ethical review board approval or exemption within 12 months of submission to the journal. We require prospective study registration. *Implementation Science* will continue to focus on publishing protocols for large scale randomised or quasi-experimental designs testing clearly defined implementation strategies, whereas *Implementation Science Communications* will consider a much broader range of study types. For example, umbrella protocols for programmes of research activity or studies where the strategies have yet to be developed should be submitted to *Implementation Science Communications*. Neither journal publishes protocols for systematic reviews or other types of evidence synthesis.

## Common reasons for rejection without review

Both journals receive a number of manuscripts that are clearly not within our scope or that fail to make a clear contribution to the field generally. We routinely desk reject these manuscripts (and offer transfer to other BMC journals). Our reject and transfer decisions largely involve four broad categories of manuscripts: 1) Studies where the primary focus is on establishing the effectiveness of clinical, health service, or population health interventions, 2) Intervention studies that are in scope, but are not hypothesis-driven or lack scientific rigor, 3) Descriptive accounts of implementation processes with little or no analytic content or linkage to the existing implementation literature, and 4) Opinion or thought pieces that are not grounded in, or do not add value to the existing implementation literature. We refer readers to a Commentary published in *Implementation Science* for more understanding of what we mean by analytic content [3].

## Core considerations for submissions

Our expectations in relation to specific types of manuscripts that fall within the scope of *Implementation Science* and *Implementation Science Communications* are summarized in Table 1. The general considerations presented apply to both journals. Alongside these requirements, there are other core issues that authors should consider when making submissions to either journal. We also encourage authors to look at the content we have been publishing in the past 12 months to gain a better sense of what is relevant for the journals.

**Table 1:** Expectations for manuscripts that fall within the scope of Implementation Science and Implementation Science Communications

	General expectations	Specific for Implementation Science	Specific for Implementation Science Communications
***Exploratory research***			
Determinants studies focused on exploring barriers and enablers to implementation	Theory / framework informed data collection and analysis with clear contribution to what is already known	Single studies not considered	Descriptive analyses or narrower disciplinary or clinical focus more likely to be considered as a Short Report
		Systematic reviews with broad applicability	
Development, pilot and feasibility studies	Focus on implementation strategies	Not considered	Comprehensive reports of strategy development
	Should submitted prior to any reporting of effectiveness		Descriptive analysis more likely to be considered as a Short Report
***Evidence synthesis***			
Systematic reviews	Recent systematic literature search (within 12 months of submission)	Analytical depth and broad generalizability	Descriptive analysis or narrower focus
Scoping reviews	Recent systematic literature search (within 12 months of submission)	Convincing rationale and demonstrable gap in the literature identified	Descriptive analysis or narrower focus
Realist reviews	Focus on implementation processes or strategies	An initial programme theory is developed and systematic steps are taken to develop, refute or refine the initial programme theory	Reviews with narrow focus
Critical interpretive synthesis	Focus on developing new theory or significant adaptations to existing ones to explain a specific issue or phenomena such as, the adverse impacts of implementation	Convincing rationale with a clear dynamic, recursive and reflexive critique of the literature	Convincing rationale with a clear dynamic, recursive and reflexive critique of the literature
Other systematic reviews of theories and frameworks	Recent systematic literature search (within 12 months of submission)	Convincing rationale and presentation of evidence that goes beyond simple descriptions of application. Reflection on what it means to apply well, what insights gained, and limitations of the theory or framework itself	Convincing rationale and presentation of evidence that goes beyond simple descriptions of application. Reflection on what it means to apply well, what insights gained, and limitations of the theory or framework itself
***Original research***			
Outcome evaluations	Focus on observable effects of implementation strategies	Randomized controlled trials and related large scale quasi-experimental designs	Broader range of evaluation designs including non-randomised and smaller scale quasi-experimental designs
Process evaluations	Related to implementation strategies and explain and contextualize the findings of main study	Theoretically informed with analytical depth and broad generalizability	Descriptive analysis or narrower focus
	Process evaluations of complex clinical or public health interventions, with a strong implementation science component considered on a case-by-case basis		
Economic evaluations	Focus on efficiency of implementation strategies	Cost-effectiveness, cost utility and cost–benefit studies	Broader range of economic designs, including cost analyses
Qualitative and mixed-methods studies	Chosen method matches with focus of the study	Orientation on theory development, use of rigorous methods, clear integration and interpretation of data sources	Studies that relate to existing theories, frameworks or concepts, seeking to guide future empirical enquiry or generating insights but with narrower focus
		Yield new theoretical insights applicable to a wide range of settings	
Quantitative observational studies	Chosen method matches with focus of the study	Study is guided by theory, use of advanced data-analysis	Broader range of designs, including more pragmatic approaches
Validation studies	Comprehensive report of development and validation of measures	New measures with rigorous psychometric analysis	Translated or adapted measures
Protocols	Received within 12 months of ethics approval and funded via the competitive review process of an established funding body	Multicentre randomized or quasi experimental designs	Broader range of study designs
	We do not consider protocols for systematic reviews or publish protocols for Type I Hybrid studies	Prospective registration	Prospective registration
***Other types***			
Capacity and capability building in implementation science	Includes evaluation or other empirical research	Programs of substantial size and continuity; longitudinal evaluation	Broader range of evaluations of educational programs
Methodology	Demonstration of methodological development	Novel methods demonstrated through concrete examples which relate to the field of implementation science	New, adapted or refined methods demonstrated through concrete examples which relate to the field of implementation science
Theories and frameworks	Convincing rationale, based on comprehensive literature review	New, elaborated theories and frameworks of broad applicability	New concepts and theoretical perspectives, and refinement to frameworks with broad or narrow applicability
Commentaries	Well-embedded in the implementation science literature	Potentially high impact and convey new or important ideas on implementation science	Contribution with moderate impact or narrower focus
	Pre-submission enquiry to Editors in Chief essential		
Debate	Based on comprehensive review of relevant literature	Potentially high impact on the field because of innovation or priority	Contribution with moderate impact or narrower focus
Short reports	Comprehensive description despite shortness	-	-

### Use of theory

We are advocates for theoretically informed research. When deploying specific theories and frameworks in studies, the rationale for use needs to be convincingly presented. Theories can be derived from the implementation science literature or from other fields but applied to implementation science. We also encourage authors to ensure that these are not applied in a superficial fashion with analysis little more than ‘structured lists of disconnected items’ [4]. Instead, we recommend in-depth engagement with selected theories and frameworks throughout the manuscript*. Implementation Science* and *Implementation Science Communications* are both increasingly reluctant to publish studies that categorize data according to a framework without offering interpretations that relate to the underlying theory or integrating findings to advance frameworks or showing novel ways to utilize frameworks to truly engage and advance the implementation process. Theories and frameworks should explicitly inform research aims and objectives, guide data collection and data analysis, shape the presentation of findings, and provide a basis for articulating the study’s contribution in the discussion section. We believe this shift in emphasis from theories as ‘products’ to theorising as a ‘process’ [3] will contribute to the advancement of knowledge in the field.

### Implementation outcomes

While we have a primary interest in the evaluation of implementation outcomes rather than clinical, patient reported, or population health effects, the outcomes of most interest to us are observable aspects of healthcare delivery, changes in practice, and or professional behaviours. We prioritize measures that focus on strategy effectiveness, adoption, cost effectiveness, penetration or reach, sustainment, and scale-up. We note that these tend to be less frequently reported in the literature [5].

Introducing interventions into health systems can have spillover effects and unintended consequences that may lead to adverse impacts, such as inequitable distribution of health, service, or implementation outcomes among different populations. This includes inequalities in access to healthcare and inequitable treatment for certain populations. We encourage authors to consider access and equity outcomes in their submissions.

In all instances, we expect authors to articulate the links between the strategies to be deployed, their impact on determinants, mechanisms of action, and the intended implementation outcomes. We do consider outcome evaluations that show negative findings or implementation strategies that had little or no impact, provided that the rationale for the chosen strategies and intended outcomes was plausible, the study design was rigorous, and the study well-conducted.

Studies where the primary focus is on establishing clinical or intervention effectiveness but where a secondary focus is on understanding acceptability and or clinical intervention fidelity are also not in scope for our purposes. Studies reporting only self-report outcomes are also unlikely to be considered by either journal.

### Methodology

We frequently receive method papers that resemble original research articles in their structure. For method papers, authors should organize their work in a way that contextualizes the research within a broader framework, illustrating the significance of the method being introduced. The background section should review the relevant literature pertaining to the method and clarify the necessity for this new approach. Following this, the paper should detail the method itself, covering its development, specific components, and potential applications. Subsequently, real research examples should be employed to demonstrate how the method can be applied in practice. The discussion section should then address the strengths and weaknesses of the method, comparing it with other similar approaches to highlight its advantages and limitations.

### Reporting

A key founding aim of *Implementation Science* was to promote efforts to improve research quality and transparency. We remain committed to this aim. We follow International Committee of Medical Journal Editors (ICMJE) recommendations for the conduct, reporting, editing, and publication of research (https://www.icmje.org/icmje-recommendations.pdf). We strongly encourage authors of all review and study types to prospectively register their protocol in publicly accessible registries.

For authorship, we adopt the ICMJE criteria for authorship. For manuscripts with large numbers of authors (e.g. 20 or more), we ask that a writing group be named that then appears in the article byline. Where studies are conducted internationally (and especially in low- and middle-income countries), we strongly recommend including at least one author from the countries of interest; all authors should meet the ICMJE criteria for authorship. We appreciate that authorship issues are complex, including cases where funding comes from one country, often a high-income country, but the research is conducted in another. We encourage research teams to consider all contributions to research, including the networks required to enable research to be conducted, and the human and social capital required for implementation research. While categories like these are not explicitly described in the ICMJE guidelines, we encourage teams to add additional elements to the statements about authorship that they consider important.

Authors of all original research manuscripts (regardless of study design) should refer to the EQUATOR network (https://www.equator-network.org/) and ensure that they complete and include an appropriate reporting checklist/s with their submission.

Both journals continue to receive submissions where implementation strategies are inconsistently labelled and/or poorly described. Without sufficient detail, it can be difficult for editors, reviewers, and readers to determine what was actually implemented or for researchers to use or replicate a strategy in other studies. The field can only develop when strategies are clearly defined and sufficiently reported to understand how they can be operationalised and, crucially, how their effects can be measured. To be of interest to either journal, this information needs to be included.

For regular research, study protocol, or systematic review manuscripts, we allow a maximum of 5500 words, debates 5000 words, and short reports 2500 words but encourage authors to use fewer words as we believe that many readers prefer concise papers. Manuscripts exceeding the journal word limits for the respective article type may be returned without review. The number and size of additional online files to a manuscript are unlimited, and these can be used to provide additional information.

Finally, we require each manuscript to include a short statement on what is already known on this topic and why this submission adds to knowledge and understanding in implementation science; this is not intended as a summary of the manuscript. This section should avoid duplication of the abstract or study findings but rather focus on how the work advances the field. These statements on added value are used by Editors to make initial assessments on whether an article merits external review.

## Conclusion

Implementation science remains a rapidly growing field internationally. Many researchers are new to the field and have varied professional and methodological backgrounds. This editorial describes the mission, scope and expectations of *Implementation Science* and *Implementation Science Communications* and aims to serve as an aid to researchers considering whether to submit to either journal.

## Data Availability

Not applicable.
